# Died—Albert Welch, M. D.

**Published:** 1886-10

**Authors:** 


DIED—At his residence in Ferris, Texas, Sept. 22, DR. ALBERT WELCH, aged 37 years.

DR. WELCH was a member of the *Physicians' Mutual Benefit Association* of Texas, and also of the *Texas State Medical Association*; having joined the latter at the Dallas meeting, last April. Assessments have been sent out to the members of the P. M. B. A. for the death benefit fund provided for in the Constitution and By-Laws of the Society, and it is hoped that members will promptly respond, as failure to pay within the time specified forfeits membership and all monies previously paid in.

